# EMDR: dispelling the false memory creation myth in response to Otgaar et al. (2022a)

**DOI:** 10.3389/fpsyg.2024.1366137

**Published:** 2024-04-16

**Authors:** Edward Callus, Eugenio Gallina, Isabel Fernandez

**Affiliations:** ^1^Clinical Psychology Service, IRCCS Policlinico San Donato, San Donato Milanese, Milan, Italy; ^2^Department of Biomedical Sciences for Health, University of Milan, Milan, Italy; ^3^Centro di Ricerca e Studi in Psicotraumatologia (CRSP), Milan, Italy; ^4^Associazione EMDR Italia, Varedo, Italy

**Keywords:** eye movement desensitization and reprocessing (EMDR), trauma, traumatic event, false memories, psychotherapy, legal

## 1 Introduction

Exposure to traumatic events can lead to diverse memory impairments such as dissociation, intrusiveness, avoidance, distortion, or recovery (Mary et al., 2020) affecting individuals' mental health, wellbeing, identity, relationships, and functioning (Giotakos, 2020). Thus, comprehending how trauma impacts memory and its assessment and treatment in clinical settings is paramount.

Otgaar et al.'s body of work, including the article under review (Otgaar et al., 2021, 2022a,b) has significantly propelled our understanding of false memories and their implications, particularly in psychological research and legal contexts. However, in the article that will be examined, the researchers claim that psychotherapy can induce false memories of trauma and that therapists can suggest clients' memories of experiences that they never had (Otgaar et al., 2022a). Despite the value of their contributions, their recent claims that psychotherapy, notably EMDR, can induce false memories of trauma warrant a closer examination. They argue that such false memories are widespread and pose risks in real-life situations, especially in legal settings, potentially compromising the credibility and justice for trauma survivors (Otgaar et al., 2022a). They also challenge the efficacy and safety of some evidence-based treatments for trauma-related disorders, such as EMDR therapy (Otgaar et al., 2022a).

In this paper, we critically examine the article by Otgaar et al. (2022a) which claims that psychotherapy can induce false memories of trauma and that therapists can suggest memories of experiences that clients never had. Specifically, Otgaar et al. (2022a) describe a unique case of therapy-induced false memories. The article recounts past cases wherein abuse was the focal point of legal proceedings. Arguments are drawn from a detailed description of an Italian case, wherein therapeutic records exhibited clear indications of suggestive treatment. The court concluded that the therapist had implanted false memories. In support of the court's determination, the authors present examples of research illustrating how false memories can be formed, including the influence of suggestion on their development.

In this opinion paper, our aim is to refute some of the authors' claims. Firstly, we will address the assertion that the reporting of abuse is not as prevalent as suggested. Secondly, we will delve into the mechanism of dissociation. Subsequently, we will analyze the methodological quality of the studies cited by the authors to support the existence of false memories. As a fourth point, we will discuss the argument that clinicians may employ suggestive techniques to induce false memories. Fifthly, we will explore the complexity and diversity of trauma and memory in real-life situations. Finally, we will highlight how Eye Movement Desensitization and Reprocessing (EMDR) is an evidence-based treatment recommended by international guidelines for addressing traumatic symptoms, emphasizing its role in facilitating healing without the induction of false memories in clients.

## 2 Discussion

### 2.1 How frequent are reports of traumatic sexual abuse?

Otgaar et al. specify that “*People who are subjected to severe trauma, such as sexual abuse, frequently talk about their experiences with, for example, the police, child protection, or friends”* (Otgaar et al., 2022a). However, it is possible to refute the fact that sexual abuse is frequently reported. While we acknowledge Otgaar et al.'s (2022b) concern regarding the potential complexities these disclosures might introduce in legal and therapeutic processes, recent data paint a contrasting picture. A very recent report by the U.S. Department of Justice estimates that only 21.5% of sexual assault and rape offenses are actually reported in 2021 (Thompson, 2022), a figure preceded by a 22.9% in 2020 (Morgan and Thompson, 2021), with a similar trend in past years (Morgan and Truman, 2019, 2020). This discrepancy underscores the complexity of trauma reporting and the myriad factors that influence a survivor's decision to come forward, aspects that are vital for both comprehending the extent of abuse and ensuring effective support.

In this regard, mentioning the Italian data from the publication “*Lives in the* Balance” (Bianchi and Moretti, 2006), which emerged from a retrospective survey conducted on behalf of the National Observatory on Childhood and Adolescence in 2005–2006. Compared to the 24% of the Italian female population aged 19–59 who reported having been victims of sexual abuse at a younger age, only 5% said they had dealt with institutions (social-health services, judicial authorities) following their disclosure.

A 2011 study by Zinzow and Thompson (2011), from Clemson University in South Carolina, aimed at identifying the resistance that encourages sexual abuse victims to remain silent. Of the 719 female students who filled out the initial questionnaire, 127 had been victims of sexual abuse; of these, 108 (85 %) had not reported the abuse they had experienced. They were asked about the reasons a victim of sexual abuse might have not chosen to disclose the experience. Among the most frequent resistances are defensive mechanisms of rationalization and denial (“*I handled it on my own*”) and minimization of the incident (“*it wasn't that bad*”).

In addition to these data, it is important to understand why, often, victims of abuse (due to feelings of shame) have difficulty exposing the traumatic event. The cause of shame in post-traumatic states is complex, but it appears that there is a multitude of overlapping factors that make shame a predominant, if not the primary, emotional experience after trauma. Research indicates that shame can be triggered by the traumatic experience itself (Budden, 2009); misplaced or inaccurate feelings of guilt or responsibility for what happened in the traumatic event (e.g., “it was my fault...” “this wouldn't have happened if I had just...”) (Wilson et al., 2006; Bhuptani and Messman, 2023); feelings of contamination and unlivability as a result of neglect or abuse, particularly in childhood (Pattison, 2000); rumination on one's own behaviors, actions, and reactions at the time of trauma (Lee et al., 2001); fear of judgment from others if they discover the trauma (Øktedalen et al., 2014) or social taboos associated with experienced trauma (e.g., childhood sexual abuse by a family member) (Banaj and Pellicano, 2020).

Pervasive feelings of shame about the incident, such as fear of being judged faulty, often lead victims blame themselves (Schwarz et al., 2017). Other factors include fear of not being believed, fear of retaliation by the perpetrator and distrust of the police and institutions in general. Another study, performed with both male and female students, reveals similar results (Sable et al., 2006).

### 2.2 Dissociation

There is, however, another factor that often represents a constant debate on false memories: the effects on memory due to dissociation, a defensive mechanism that the brain puts in place to protect itself from the destructive effects of traumatic experience. Otgaar et al. (2022a) acknowledge dissociation but frame it in a manner that we believe simplifies its origins and connection to trauma. In response, we aim to highlight the nuanced understanding of dissociation as a psychobiological reaction, supported by extensive neurobiological evidence (Loftus, 1993; Laney and Loftus, 2005; Brand et al., 2017). This understanding not only challenges the reductionist view but also enriches our appreciation for the sophisticated ways in which the human mind copes with trauma. Dissociation as a Response to Trauma is understood as a psychobiological reaction that can emerge in response to highly distressing and/or traumatic situations: escape of the mind when physical escape is not possible (Nijenhuis et al., 1998; Vermetten et al., 2007).

Neurobiological findings that contributed to the introduction of the PTSD-D diagnosis (Lanius et al., 2014) have also provided support for conceptualizations of dissociation as a trauma-related response. Being able to discuss neurobiological findings linked to dissociation can help facilitate understanding of dissociation and underscore the connection between trauma and dissociation.

Recent research using objective measures (such as functional magnetic resonance imaging and skin conductance) has found that trauma-related depersonalization and derealization are associated with overmodulation of emotional responsiveness (Sierra and Berrios, 1998). The overmodulation model is also consistent with findings indicating an inverse relationship between the severity of dissociation and cortisol reactivity (Simeon et al., 2007), as well as reduced skin conductance and increased response latency in patients with chronic depersonalization when exposed to unpleasant images (Sierra et al., 2002). Regarding dissociative identity disorder's (DID) dissociative self-states (DSS), research has demonstrated that different DSSs show different neurobiological responses to their own traumatic scripts. Specifically, when individuals are tested while in a self-state that endorses having experienced the trauma as a personal event, they exhibit the typical emotional and physiological hyperarousal expected in PTSD. However, if they undergo brain scans or other types of testing while in a self-state that does not confirm traumatic events as autobiographical, they do not show the classic signs of heart rate variability, such as increased heart rate, systolic blood pressure, or the brain network patterns indicative of emotional hyperarousal. Instead, they exhibit a pattern of emotional overmodulation, including increased activation in the medial prefrontal cortex, insula, and amygdala (Tsai et al., 1999; Reinders et al., 2003, 2006, 2012, 2014; Lanius et al., 2010; Schlumpf et al., 2013).

In this regard, it is of utmost importance to mention two important articles in which the same authors report, with incontrovertible evidence by means of functional and structural magnetic resonance imaging, the presence in clients confronted with their own traumatic memory of neurobiological alterations in the amygdala and prefrontal cortex associated with dissociative symptoms demonstrating its presence as a protective factor against the distress caused by revisiting the trauma (Lanius et al., 2010; Nardo et al., 2013).

Dissociative experiences serve an avoidance function. Experiences of derealization, depersonalization, gaps in awareness, and amnesia can function to distance a person from a negative emotional experience in the present moment. Dissociative experiences can also prevent cognitive awareness or divert attention away from stimuli or events that would be distressing, which can be considered a form of experiential avoidance (Carlson et al., 2012).

In addition to the scientific confirmation, according to the ICD-11 and the DSM-5, dissociation is the involuntary disruption or discontinuity of the normal integration of various aspects of psychological functioning, such as identity, memory, perception, or behavior (American Psychiatric Association, 2013; World Health Organization, 2019).

### 2.3 Reliance on questionable methodology

Otgaar et al.'s (2022b) article also relies on a methodology to study false memories and trauma that we find questionable. While the exploration of these themes is undoubtedly valuable, we posit that the experimental conditions described do not fully encapsulate the complexities of real-life trauma experiences and memory dynamics. Muschalla and Schönborn (2021) highlight the diversity and potential for inducing false beliefs or memories under experimental conditions, yet they also caution about the heterogeneity and limitations of these findings (Muschalla and Schönborn, 2021). This critique is not to diminish the importance of laboratory research but to advocate for a broader methodological approach that can more accurately reflect and address the lived experiences of trauma survivors.

Another article discusses the role of mental imagery in memory distortion for traumatic events and notes that there are significant methodological limitations to keep in mind when evaluating all laboratory-based research on traumatic memory (Strange and Takarangi, 2015). The authors argue that although laboratory research can provide critical insights because of tightly controlled experimental designs, it is frequently a poor analog for an event that meets the criteria described.

These references suggest that while laboratory experiments can provide some insights into the nature of false memories and trauma, they have limitations in terms of their ecological validity and generalizability. They may not accurately reflect the complexity and diversity of trauma and memory in real-life situations, underscoring the need for methodological approaches that encompass the multifaceted nature of human experiences with trauma.

### 2.4 Distorted and biased view on clinicians

Otgaar et al. (2022b) article also has a distorted and biased view on clinicians who work with trauma survivors and their memories. The article suggests that clinicians are naive and uncritical about the possibility of dissociation and false memories, and they are accused of using suggestive techniques that induce false memories in their clients.

However, this portrayal is overly simplistic and fails to recognize the depth of understanding and the critical approach clinicians apply to their work. Firstly, clinicians who work with trauma survivors and their memories are not naive or uncritical about the possibility of dissociation and false memories. They are deeply aware of and critical about the complexity and diversity of trauma and memory in real-life situations. They follow empirical evidence, clinical experience, ethical principles, and professional standards that guide their assessment and treatment of trauma survivors and their memories (Malacrea et al., 2022).

Furthermore, the recognition and endorsement of EMDR by reputable mental health organizations, including the American Psychological Association (APA) and the WHO, stand as testament to its effectiveness. These endorsements not only validate the clinical utility of EMDR but also reflect its acceptance within the mental health community at large. EMDR has been the subject of numerous research studies and has been recognized as an efficient and effective treatment for PTSD in civilian populations by the American Psychological Association (APA). The International Society for Traumatic Stress Studies (ISTSS) further supports EMDR, deeming it an effective treatment guideline for complex PTSD in adults (Cloitre et al., 2012). Additionally, entities, such as the Clinical Resource Efficacy Team of the Northern Ireland Department of Health, the Quality Institute Health Care CBO/Trimbos Institute, the French National Institute of Health and Medical Research, and the American Psychiatric Association, have considered EMDR as an elective treatment for PTSD along with CBT.

Secondly, it is critical to note that clinicians who work with trauma survivors and their memories do not employ suggestive techniques that induce false memories in their clients. Clinicians use evidence-based treatments for trauma-related disorders, such as EMDR therapy, that respect the autonomy and competence of their clients. Clinicians facilitate and monitor the natural information processing system of their clients, not influence or manipulate it (Shapiro, 2001).

Lastly, clinicians are trained and informed by reputable and credible sources that reflect the current state of knowledge and practice in the field of psychotraumatology. This commitment to ongoing education and professional development ensures they remain proficient in assisting trauma survivors, further enhancing their skills and understanding (Malacrea et al., 2022).

### 2.5 Subversion of the conclusions of a reliable study

Otgaar et al. (2022b) article also misinterprets the conclusions of a reliable study by Goodman et al. (2018) that examined the memories of subjects who had actually experienced traumatic experiences during childhood. Otgaar et al. (2022a) use the results of this study to support their claim that a true traumatic memory would be reported accurately and vividly, implying that less vivid reenactments would likely be attributable to false memories induced by suggestion. However, this interpretation is inaccurate and misleading, as it overlooks other important findings and implications of Goodman et al. (2018) study.

Firstly, Otgaar et al. (2022a) overlook that Goodman et al. (2018) also found that traumatized subjects confidently recalled the core of events, i.e., the abuse they experienced, but that details naturally tended to blur over time. This finding suggests that memory accuracy and vividness are not fixed or linear measures, but rather depend on various factors, such as the type and intensity of trauma, the type and relevance of details, the time elapsed since the trauma, etc. (Goodman et al., 2018).

Secondly, Otgaar et al. (2022a) misinterpret the study by Goodman et al. (2018) where the authors state that 30% of their subjects recovered memories of traumatic experiences after a prolonged period in which they had no memory of them, mistakenly attributing this to the creation of memories through suggestion. Instead, this finding simply suggests that dissociation and recovery of traumatic memories are real and common phenomena among trauma survivors.

Finally, Otgaar et al. (2022b) fail to acknowledge that Goodman et al. (2018) also found that none of their subjects recalled the suggested false events that were implanted by the interviewers during their experiment. This finding suggests that trauma survivors are not easily susceptible to false memories induced by suggestion, especially when they have strong and consistent memories of their abuse (Goodman et al., 2018).

### 2.6 Regarding EMDR therapy

Otgaar et al.'s (2022a) article also questioning EMDR therapy, an evidence-based treatment for trauma-related disorders, by claiming that it involves suggestive techniques that induce false memories. They argue that EMDR therapy causes a deflation of imagination, or a facilitation of memory retrieval caused by eye movements that could potentially lead to false memories. Furthermore, they contend that EMDR therapy is not supported by any robust neurobiological foundation.

However, this attack is unfounded and misleading. Firstly, EMDR therapy does not involve suggestive techniques that induce false memories. On the contrary, EMDR therapy follows a very structured protocol that requires the therapist to refrain from intervening, speaking, or suggesting anything to the client during the reprocessing of traumatic memories (Shapiro, 2001). The therapist's role is to facilitate and monitor the client's natural information processing system, not to influence or manipulate it (Shapiro, 2001). EMDR therapy is applied only on episodic memories that the client is able to describe before starting the treatment, not on repressed memories or memories that are not accessible to consciousness (Shapiro, 2001).

Secondly, EMDR therapy is supported by a vast and growing body of literature and practice that demonstrates its efficacy and safety for various psychological disorders (WHO, 2013; Bisson et al., 2019). EMDR therapy is based on empirical evidence, clinical experience, ethical principles, and professional standards that respect the complexity and diversity of trauma and memory in real-life situations (Malacrea et al., 2022). EMDR therapy does not induce or implant false memories in clients; rather, it facilitates and monitors their adaptive information processing using eye movements as a stimulation technique (Baek et al., 2019).

Thirdly, EMDR therapy is supported by neuroimaging studies that demonstrate its solid neurobiological foundation. These studies show how EMDR therapy contributes to the adaptive information processing of traumatic memories by stimulating various brain regions and structures that are involved in memory encoding, storage, and retrieval.

EMDR therapy helps to normalize their activity by facilitating the migration, processing, contextualization, integration, consolidation, or reconsolidation of traumatic memories (Harper et al., 2009; Pagani et al., 2011, 2012, 2017; Landin-Romero et al., 2018; Baek et al., 2019; Mattera et al., 2022).

## 3 Conclusion

The discussion surrounding traumatic memories, dissociation, and the role of psychotherapy in memory recall is complex and multifaceted. This comprehensive analysis has delved into various aspects, including the frequency of reporting traumatic sexual abuse, the impact of dissociation on memory, the reliability of a single case study, the methodology employed in studying false memories, the portrayal of clinicians, the interpretation of reliable studies, and the critique of EMDR therapy.

Starting with the frequency of reporting traumatic sexual abuse, it is evident that a significant discrepancy exists between reported cases and actual instances. While some argue that a substantial number of trauma survivors come forward and discuss their experiences, empirical data suggests that a considerable percentage remains undisclosed, highlighting the intricate nature of reporting sexual abuse. Factors such as shame, fear of judgment, and distrust of authorities contribute to the underreporting phenomenon, making it imperative to consider the multifaceted reasons survivors choose to remain silent.

Dissociation emerged as a crucial element in the discussion, challenging the notion that it is merely a myth propagated by false memory proponents. Research, including neurobiological findings, supports the existence of dissociation as a response to trauma. This defensive mechanism serves as a psychobiological reaction to distressing situations, emphasizing its role in protecting the mind when physical escape is unattainable. The complexity of dissociative experiences, including depersonalization and derealization, was explored, shedding light on their potential functions in avoiding negative emotional experiences.

The critique of Otgaar et al.'s (2022a) single case study and their reliance on questionable methodology exposed potential limitations in their conclusions. A nuanced examination of trauma survivors and their memories requires a broader perspective than what a singular case can provide. Additionally, the methodology employed in laboratory experiments, while informative, may lack ecological validity and struggle to capture the intricacies of real-life trauma and memory.

A distorted view of clinicians was addressed, emphasizing that professionals working with trauma survivors are neither naive nor uncritical about dissociation and false memories. EMDR therapy, an evidence-based treatment, faced unwarranted skepticism. The unfounded claims that EMDR involves suggestive techniques that induce false memories were debunked, highlighting the structured and ethical nature of this therapeutic approach. Moreover, EMDR therapy's extensive empirical support, safety, and neurobiological foundation were underscored, countering the unsubstantiated criticisms.

In summary, the examination of these topics calls for a balanced and nuanced approach. Trauma survivors' experiences are intricate, influenced by psychological, emotional, and societal factors. Dissociation, rather than being dismissed as a myth, is acknowledged as a genuine response to trauma, supported by neurobiological evidence. Criticisms of psychotherapy, particularly EMDR, should be scrutinized within the framework of robust empirical evidence and ethical considerations. The path forward in trauma research and therapy necessitates collaborative efforts, open dialogue, and a commitment to exploring and addressing the complexities of trauma and memory with empathy, rigor, and an interdisciplinary approach. The evolving landscape of trauma research underscores the need for ongoing dialogue and exploration, respecting the complexity inherent in the study of memory and its relation to traumatic experiences.

## Author contributions

EC: Writing – original draft, Writing – review & editing. EG: Writing – original draft, Writing – review & editing. IF: Writing – original draft, Writing – review & editing.
